# Transhiatal esophagectomy as a treatment for locally advanced adenocarcinoma of the gastroesophageal junction: postoperative and oncologic results of a single-center cohort: THE for locally advanced GEJC

**DOI:** 10.1186/s12957-022-02537-x

**Published:** 2022-03-06

**Authors:** Hélène Meillat, Vincent Niziers, Christophe Zemmour, Jacques Ewald, Jean-Philippe Ratone, Slimane Dermeche, Jérôme Guiramand

**Affiliations:** 1 Department of Digestive Surgical Oncology, Institut Paoli Calmettes, 232 Boulevard de Sainte Marguerite, 13009 Marseille, France; 2 Department of Clinical Research & Investigation, Biostatistics & Methodology Unit, Paoli Calmettes Institute, Aix Marseille University, INSERM, IRD, SESSTIM, Marseille, France; 3 Digestive Endoscopy Unit, Paoli Calmettes Institute, Marseille, France; 4 Department of Medical Oncology, Institut Paoli Calmettes, Marseille, France

**Keywords:** Esophageal neoplasms, Neoadjuvant therapy, Esophagectomy, Survival

## Abstract

**Background and purpose:**

To report the postoperative and oncological outcomes of transhiatal esophagectomy for locally advanced cancer of the gastroesophageal junction.

**Methods:**

Medical records of 120 consecutive patients who underwent transhiatal esophagectomy for locally advanced cancer of the gastroesophageal junction with curative intent after neoadjuvant treatment between February 2006 and December 2018 at our center were reviewed.

**Results:**

All patients received either chemotherapy (46.7%) or chemoradiation (53.3%). The 90-day mortality and overall morbidity rates were 0.8% and 56.7%, respectively. Respiratory complications were the most common (30.8%). Anastomotic leakage occurred in 19 patients (15.8%), who were treated by local wound care (*n* = 13) or surgical drainage (*n* = 6). Recurrent laryngeal nerve injury occurred in 12 patients (9.9%). The median length of hospital stay was 15.5 days. The rate of R0 resection was 95.8%, and the median number of nodes removed was 17.5. Over a median follow-up of 77 months, the rate of recurrence was 40.8%, and the overall survival rates at 1, 3, and 5 years were 91%, 75%, and 65%, respectively. The median survival time was not reached. In multivariate analysis, disease stage was the only independent significant prognostic factor.

**Conclusions:**

Transhiatal esophagectomy is a safe and effective procedure with good long-term oncological outcomes for locally advanced tumors after neo-adjuvant treatment. It can be recommended for all patients with cancer of the gastroesophageal junction, regardless of the Siewert classification, tumor stage, and comorbidities.

## Introduction

Cancer of the gastroesophageal junction (CGEJ) remains a significant clinical problem with an increasing incidence [1] and is associated with a poor long-term prognosis [2]. Surgical resection remains the mainstay of curative treatment; however, multiple randomized trials have established neoadjuvant treatment as the standard approach in the management of patients with locally advanced tumors (T3, T4, or node positive) [3, 4]. Consensus on the type of surgery that offers the optimal chance of a cure varies worldwide, especially in relation to the extent of lymphadenectomy. In France, transthoracic esophagectomy (TTE) is recommended for achieving oncological resection with radical en-bloc dissection [5], which could provide a potential long-term survival benefit over transhiatal esophagectomy (THE) [6, 7]. Studies on which this recommendation is based enrolled only patients who did not receive neoadjuvant therapy and showed similar overall 5-year survival rates in both groups (36% vs. 34%) in recently updated results [8, 9].

The main argument in favor of TTE is the supposedly higher number of lymph nodes retrieved than with THE, as a minimum of 23 nodes harvested is currently recommended [7, 9]. Indeed, detractors argue that THE does not allow an optimal lymphadenectomy, unlike the thoracic approach. However, the impact of an extended lymphadenectomy on survival is still debated, because studies on this matter combined adenocarcinoma and squamous cell carcinoma, despite their different tumoral and biologic behavior [10–15], and mixed patients with and without neoadjuvant treatment [7, 10, 16]. Moreover, it appears that the number of lymph nodes examined decreases after neoadjuvant treatment with a modified distribution of metastases, predominating in the abdominal and peritumoral sites [17], with the number resected being equal using the two approaches.

The main advantage of THE is reduced morbidity, especially cardiorespiratory complications.

Consequently, there is no strong evidence to support the use of one technique over the other. The purpose of this study was to report our oncological results with THE for locally advanced CGEJ after neoadjuvant treatment.

## Methods

### Patient selection

Between February 2006 and December 2018, 120 consecutive patients with locally advanced CGEJ, according to the Siewert classification [18], underwent surgical resection with curative intent at the Institut Paoli Calmettes (Marseille, France), and their medical records were subsequently reviewed. Patients with metastatic disease or poor general status precluding extensive surgery were excluded. All patient data were entered prospectively into a clinical database, which was approved by both the Institutional Review Board and the ethics committee (N° IPC 2019-057). Informed consent was obtained from all patients before surgery, and the study protocol was conducted in accordance with the 1989 World Medical Association Declaration of Helsinki.

### Preoperative assessment

Initial and preoperative evaluations included upper gastrointestinal endoscopy with biopsy, endoscopic ultrasonography (EUS), thoracic and abdominal computed tomography (CT), and tumor markers [carbohydrate antigen 19.9 (CA 19-9) and carcinoembryonic antigen (CEA)]. Staging laparoscopy and positron emission tomography (PET) were not routinely performed.

The included patients received neoadjuvant treatment in accordance with previous recommendations [3, 4]. At our institution, we standardized this treatment according to the tumor location; patients received chemotherapy (Siewert II and III) or chemoradiation (CRT) (Siewert I) if the tumor was classified as T3/T4 and/or N+ based on EUS findings. In case of doubt or impossibility of determining the precise location of the lesion at the time of EUS, the choice of treatment was based on CT findings; CRT was indicated when the tumor was mainly located in the thorax, and chemotherapy was indicated when the tumor was mainly located in the abdomen or at the junction.

Perioperative chemotherapy agents evolved throughout the study period and consisted of platinum and fluorouracil in combination with epirubicin [4, 19] or docetaxel [20], according to recommendations and the data in the literature.

Neoadjuvant CRT consisted of a total dose of 45 Gy delivered according to the technique described by Bosset et al. [21] combined with concurrent chemotherapy (platinum-based chemotherapy and 5-FU).

### Surgery

The same surgeon performed all resections, with the help of a second surgeon during the cervical phase. Surgery was performed through the open bi-subcostal approach or laparoscopically. Patients with Siewert III tumors underwent classical total gastrectomy [5] and were consequently not included in the present study. However, THE was performed when the intrathoracic location of the upper pole of the tumor did not allow for total gastrectomy with gastroesophageal anastomosis (in case of large tumor volume or hiatal hernia). Tissue and lymph nodes along the common hepatic artery, celiac trunk, and top of the pancreas and those from the splenic artery to the spleen were removed en-bloc, along with lymph nodes along the lesser curve of the stomach, cardia, and specimen. A wide splitting of the esophageal hiatus (with an incision in the right diaphragmatic crus) allowed for dissection of the lower mediastinum with circumferential removal of the fat pad around the thoracic esophagus as far as the carina under visual control (the aorta was viewed backward, the mediastinal pleura were resected on both sides, and the pericardium was viewed forward, serving as the margins for dissection). After left-sided mobilization and retrosternal dissection of the cervical esophagus and upper mediastinum without lymphadenectomy, blind dissection was not necessary. Gastrointestinal continuity was re-established using a 5- to 10-cm-wide gastric tube that was vascularized by the right gastro-epiploic artery and positioned within the posterior mediastinum via cervical end-to-end anastomosis with manual interrupted stitches (PDS 4.0).

Mediastinal and cervical drains were systematically inserted, but chest tubes were not routinely used. Enteral nutrition was supplied through a feeding jejunostomy from day 1, and the patients were allowed oral feeding after the nasogastric tube was removed between days 3 and 7. A water-soluble oral contrast study was conducted only on the suspicion of dehiscence of the anastomosis.

### Histological analysis

All tumors were staged according to the seventh edition of the American Joint Commission on Cancer Staging Manual’s (AJCC 7) criteria for esophageal cancer by an experienced pathologist [22]. The surgeon identified all groups of removed lymph nodes intraoperatively and submitted them as separate specimens for counting and examination according to their location. A negative margin (R0) was defined as a clear circumferential and longitudinal margin [22].

### Study parameters

Postoperative mortality was defined as death occurring within 90 days after surgery. Postoperative morbidity was graded according to the Clavien-Dindo classification [23].

Delayed complications were defined as any complication that occurred more than 1 month after THE and included principally recurrent laryngeal nerve injury and benign anastomotic stricture.

### Patient follow-up

After discharge, patients were routinely followed up at the outpatient hospital at 1 month postoperatively. Considering their recovery status, patients were offered adjuvant therapy based on the same regimen as that for preoperative chemotherapy, regardless of the results of pathological examination. In case of preoperative radiotherapy, no adjuvant treatment was offered. A physical examination, CT, and tumor marker analysis (CA 19.9 and CEA) were performed at 4-month intervals for 2 years and twice a year for 5 years or until death.

### Statistical analysis

All statistical analyses were performed at the significance level of α = 0.05 using SAS® 9.4 software (SAS Institute, Cary, NC). Categorical variables were summarized as frequencies (%) calculated based on available data and quantitative variables as medians (range). Continuous data were compared using the Mann-Whitney *U* test and categorical data using the Fisher exact test.

Overall survival (OS) and disease-free survival (DFS) were defined from the date of operation. Patients without events were right-censored at the date of their most recent follow-up. Survival endpoints were estimated using the Kaplan-Meier method. For administrative reasons, the OS and DFS data were censored after 10 years of follow-up.

Multivariate Cox models that included the ASA score, Siewert classification, number of harvested lymph nodes, and pTNM stage as independent covariates were analyzed. The associated hazard ratios (HRs) were estimated with their Wald’s bilateral confidence intervals and *p*-values.

## Results

### Demographic data

Demographic details of the 120 patients are shown in Table 1. More than 90% of the patients had a T3 esophageal adenocarcinoma at diagnosis, with suspected lymph node involvement in 96 patients (80%). Fifty-six patients (46.7%) received preoperative chemotherapy and 64 (53.3%) received preoperative CRT.

**Table 1:** Demographics of the 120 patients with CGEJ

Clinicopathologic factor	Overall cohort, no. (%)
**Sex**	
- Male	105 (87.5%)
- Female	15 (12.5%)
**Age**, years^a^	64 (26-81)
**BMI**, kg/m^2a^	25 (15.6-37)
**Malnutrition**	54 (45%)
**ASA score**	
- 1	15 (12.5%)
- 2	92 (76.7%)
- 3	13 (10.8%)
**One or more comorbidities**	
- Cardiac	54 (45%)
- Vascular	17 (14.2%)
- Pulmonary	22 (18.3%)
- Diabetes mellitus	8 (6.7%)
**Smoking history**	84 (70%)
**Reflux history**	28 (23.3%)
**Preoperative Siewert classification**	
- I	63 (52.5%)
- II	54 (45%)
- III	3 (2.5%)
**Pretreatment T stage**	
- cT2	6 (5%)
- cT3	110 (91.7%)
- cT4	4 (3.3%)
**Pretreatment N stage**	
- cN0	24 (20%)
- cN+	96 (80%)
**Preoperative treatment**	
- Chemotherapy	56 (46.7%)
- Chemo-radiotherapy	64 (53.3%)

*ASA* American Society of Anesthesiologists, *BMI* body mass index, *CGEJ* locally advanced cancer of the gastroesophageal junction, *c* clinical stage

^a^Expressed as median (range)

### Surgery and postoperative course

The intraoperative and postoperative outcomes are listed in Table 2. Two patients (1.6%) required thoracotomy: one to repair a wound of the left main bronchus and one to repair an aortic wound. The overall 90-day morbidity and mortality rates were 56.7% and 0.8%, respectively. One patient had unexplained circulatory collapse during the surgical intervention and died on postoperative day 25 due to toxic shock.

**Table 2:** Intraoperative and postoperative outcomes

Operative time, min^a^	240 (180–600)
Intraoperative blood loss, mL^a^	200 (0–3000)
Red cell transfusion	16 (13.3%)
90-day mortality	1 (0.8%)
90-day morbidity (Clavien-Dindo)	68 (56.7%)
Grade I/II	33 (27.5%)
Grade IIIa/IIIb	23 (19.2%)
Grade IV	11 (9.2%)
Grade V	1 (0.8%)
Clinical anastomotic leak	19 (15.8%)
Surgical drainage	12 (10%)
Respiratory	38 (31.7%)
Respiratory failure	11 (9.2%)
Pneumonia	25 (20.8%)
Pleural drainage	21 (17.5%)
Mediastinitis	6 (5%)
Chylothorax	3 (2.5%)
Bleeding	5 (4.2%)
Recurrent nerve injury	12 (10%)
Others:	
Atrial fibrillation	12 (10%)
Reintervention	15 (12.5%)
Time to discharge, day^a^	15.5 (10–120)
Readmission	10 (8.4%)

^a^Expressed as median (range)

The anastomotic failure rate was 16% (*n* = 19). All patients who developed a fistula had a favorable outcome within 2–4 weeks, with a median oral refeeding time of 14 days (range 7–55 days). However, the occurrence of a fistula significantly prolonged the median length of hospital stay (36.3 vs. 18.6 days, *p* < 0.01).

The overall respiratory complication rate was 31.7% (*n* = 38). The severe respiratory complication rate was 20.8% (*n* = 25), including pleural drainage alone (Clavien-Dindo 3a; *n* = 14) and reintubation for respiratory failure (Clavien-Dindo 4, *n* = 11).

The benign anastomotic stricture rate was significantly higher in patients who experienced a fistula (42.1% vs. 6.9%, *p* < 0.01). All patients with benign strictures were successfully treated by endoscopic dilatation at a median of 3.2 sessions.

Recurrent laryngeal nerve injury occurred in 12 patients (9.9%). The hoarseness was usually transient due to vocal cord paresis and resolved within 2–12 weeks. Eight patients with persistent dysphonia (6.6%) required cord medialization.

### Histopathological analysis

Results of the histopathological analysis of the operative specimens are summarized in Table 3. Curative resection (R0) was achieved in 115 patients (95.8%). Radial margins were involved in 4 patients with T4a or T3N+ tumors on pathological staging. A median of 17.5 lymph nodes were dissected from each specimen, and lymph node metastases were found in 53 patients (44.2%). The most frequent sites of nodal metastases were the celiac axis (*n* = 36), mediastinum (*n* = 23), and lesser curvature of the stomach (*n* = 22), showing no significant difference according to the Siewert classification. The mean lymph node ratio was 0.26 (range 0.04–0.79, median 0.15). In 22 patients (18.3%), there was a complete pathological response to neoadjuvant treatment (16 after CRT and 6 after chemotherapy).

**Table 3:** Pathological findings

	Overall cohort, no. (%)
Tumor classification	
pT0-Tis	24 (20%)
pT1	15 (12.5%)
pT2	25 (20.8%)
pT3	53 (44.2%)
pT4	3 (2.5%)
Tumor size, mm^a^	30 (0-160)
Nodes classification	
pN0	67 (55.8%)
pN1	25 (20.8%)
pN2	15 (12.5%)
pN3	13 (10.8%)
Stage	
Stage 0	22 (18.3%)
Stage I	13 (10.8%)
Stage II	34 (28.3%)
Stage III	36 (30%)
Stage IV	15 (12.5%)
Number of examined LN^a^	17.5 (6-36)
Number of mediastinal examined LN	4 (0-16)
Number of positive lymph nodes^a^	3 (1-23)
Perineural invasion	22 (18.3%)
Vascular embolism	20 (16.7%)
Tumor differentiation	
Poor	16 (13.3%)
Intermediate	37 (30.8%)
Well	41 (34.2%)
Mucinous	2 (1.7%)
Sterilized tumor	24 (20%)
Margin status	
R0	115 (95.8%)
R1	5 (4.2%)

*LN* lymph node, *p* post-therapeutic classification

^a^Expressed as median (range)

### Survival and recurrence

**Fig. 1:**
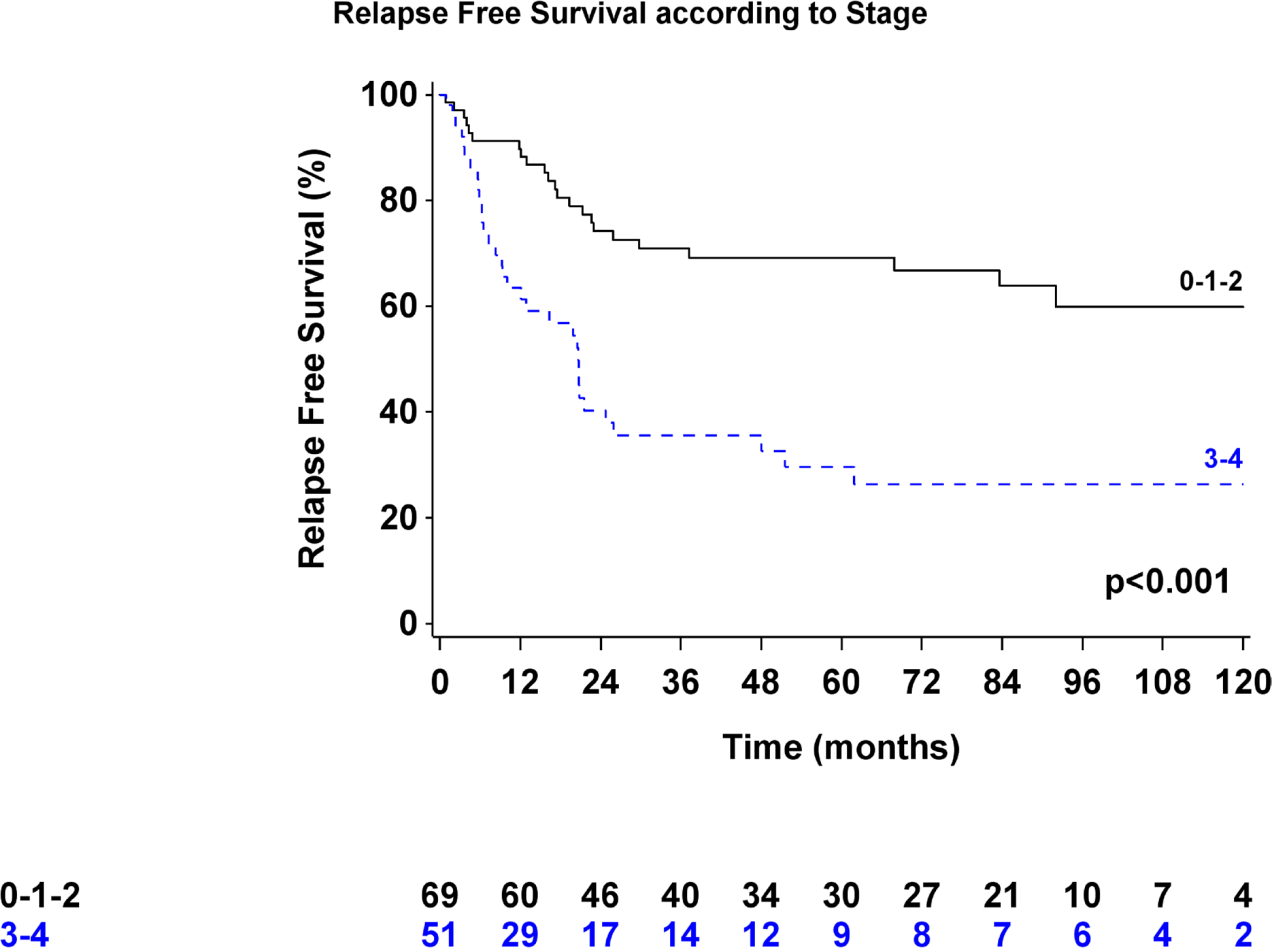
Kaplan-Meier curve for disease-free survival after THE according to pathologic stage

At a median follow-up of 77.8 months, the overall 1-, 3-, and 5-year survival rates of all patients were 91% (95% confidence interval 84%-95%), 75% (66%-83%), and 65% (54%-74%), respectively (Fig. 1). The median OS time was not reached. Forty-four (36.7%) patients received adjuvant treatment (chemotherapy: 29 patients, CRT: 15 patients).

**Fig. 2:**
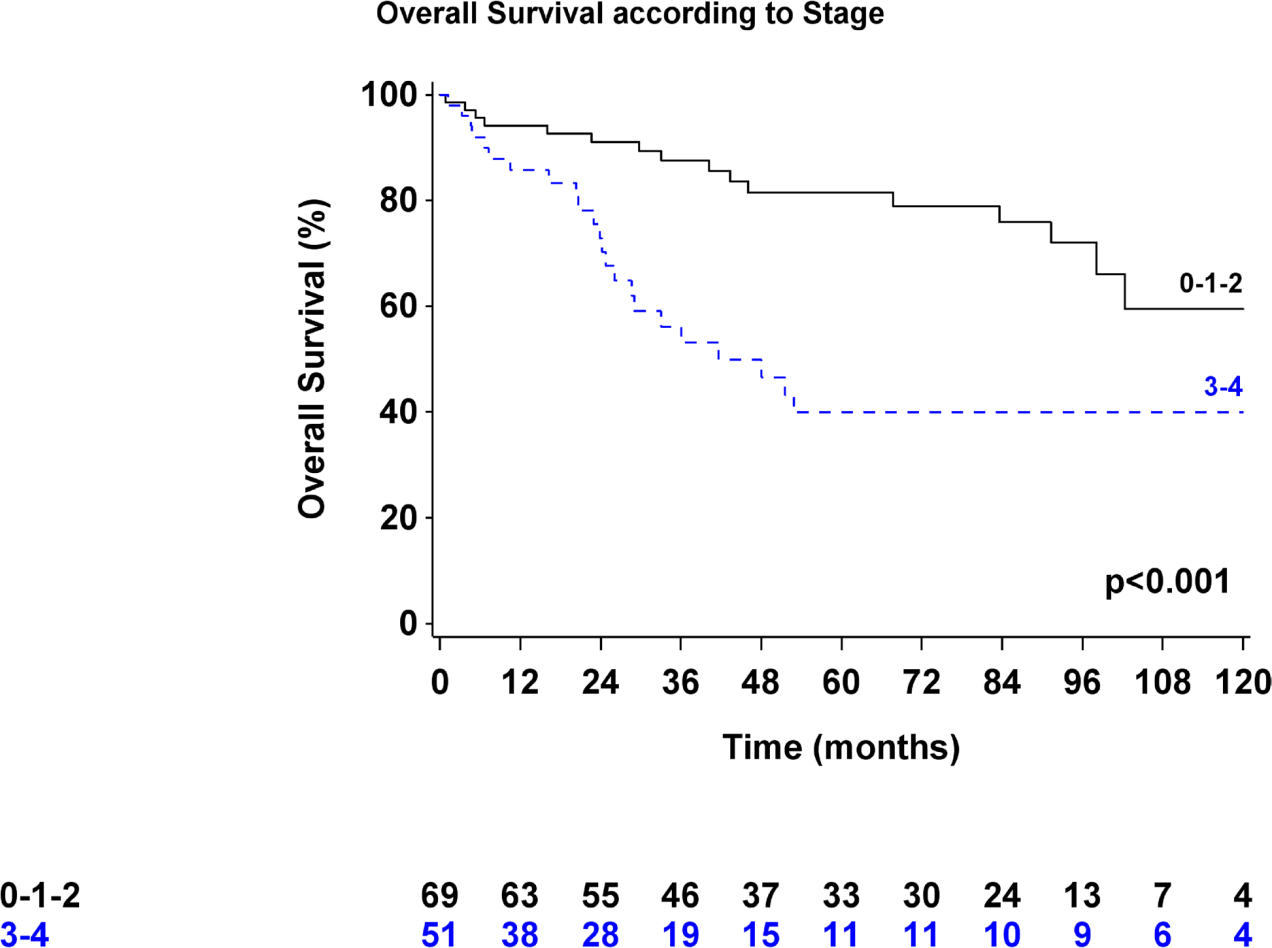
Kaplan-Meier curve for overall survival after THE according to pathologic stage

Forty-nine patients (40.8%) developed recurrent disease in the peritoneum (*n* = 14), lungs (*n* = 11), liver (*n* = 10), lymph nodes (*n* = 9), and anastomotic site (*n* = 5). DFS at 3 years was 56% (47–65%) (Fig. 2).

The sites of recurrent lymph nodes were the supraclavicular (*n* = 3), cervical (*n* = 1), upper mediastinum (*n* = 5), and lumboaortic (*n* = 5) regions. These sites were associated with metastases in other locations in 50% of cases. There was no correlation between the type of preoperative treatment and site of lymph node recurrence.

Multivariate analyses showed that only TNM stage was independently associated with poor prognosis (Tables 4 and 5). The Siewert classification, ASA score, and number of harvested lymph nodes were not significantly related to OS or DFS.

**Table 4:** Multivariate analysis of the overall survival

	Patients	Events, no. (%)	HR [95% CI]	*p*
ASA score				
1–2	107	36 (33.6%)		
3–4	13	3 (23.1%)	0.87 [0.25–3.02]	0.82
Siewert classification				
Siewert I	63	24 (38.1%)		
Siewert II–III	57	15 (26.3%)	0.51 [0.25–1.07]	0.07
TNM stage				
Stage 1–2	69	16 (23.2%)		
Stage 3–4	51	23 (45.1%)	3.94 [1.92–8.09]	< 0.001
Number of harvested LNs				
< 15	42	18 (42.9%)		
≥ 15	78	21 (26.9%)	0.6 [0.3–1.2]	0.15

*HR* hazard ratio, *CI* confidence interval, *p* Wald’s test *p* value for significance, *LN* lymph node

**Table 5:** Multivariate analysis of the disease-free survival

	Patients	Events, no. (%)	HR [95% CI]	*p*
**ASA score**				
1–2	107	51 (47.7%)		
3–4	13	5 (38.5%)	0.89 [0.34–2.34]	0.81
**Siewert classification**				
Siewert I	63	30 (47.6%)		
Siewert II–III	57	26 (45.6%)	0.75 [0.41–1.37]	0.35
**TNM stage**				
Stage 1–2	69	23 (33.3%)		
Stage 3–4	51	33 (64.7%)	3.39 [1.88–6.12]	< 0.001
**Number of harvested LN**				
< 15	42	22 (52.4%)		
≥ 15	78	34 (43.6%)	0.72 [0.39–1.31]	0.28

*HR* hazard ratio, *CI* confidence interval, *p* Wald’s test *p* value for significance, *LN* lymph node

## Discussion

Our monocentric series showed that THE was a safe technique and provided good oncologic outcomes in patients with locally advanced CGEJ.

### Postoperative course

Despite a high morbidity rate (57.1%), we observed a dramatically low mortality rate (1.2%) compared with other esogastric surgical procedures [12, 24–27]. We made the choice to use the Clavien-Dindo classification, as recommended by the esophagectomy complications consensus group [28], considering all complications until postoperative day 90. However, the widely varying definitions for complications used make comparisons between studies difficult. Minor complications are scarcely described in esophageal surgery studies because they are very secondary to life-threatening complications but they represent half of the complications identified in our study.

Patients who experienced anastomotic leakage in our series developed moderate sepsis with isolated cervical suppuration but no mediastinis. This non-severe event was managed by reopening of the cervical wound and local wound care, and all patients showed favorable outcomes.

The overall respiratory complication rate (31.6%) was higher than that in other studies [7, 14], but only one-third of these complications were major complications. The other two-thirds only required physiotherapy, antibiotics, or short-term pleural drainage, with no major repercussions.

Unlike TTE, we did not perform routine thoracic but only active abdominal drainage placed in the lower mediastinum. However, edema after neoadjuvant CRT, chylothorax, and/or large bilateral mediastinal pleura resection may cause fluid to accumulate into the thorax and indicate postoperative pleural drainage (17.5% in our series).

### Oncologic results

Tumor stage, surgical resection margin, and lymph node status are the most important predictors of outcome in patients with esophageal cancer [11, 25]. Opponents of THE claim that the performance of a more extended resection to the upper mediastinum improves the latter two criteria, and thus long-term survival [7, 26]. Their conclusions are based on studies that included all esophageal tumors, regardless of their location or pathological features, and in the absence of neoadjuvant treatment.

Some authors suggest that radical TTE reduces the likelihood of margin involvement, particularly in patients with T3 to T4 tumors [6, 26]. Although it is accepted that THE cannot offer the same access to the mediastinum, the lack of standardization in the volume of periesophageal tissue resection [27, 29] is a confounder that undermines studies assessing surgical radicality. Despite the high portion of advanced tumors at diagnosis in our series, the rates of microscopically positive (R1) resection margins (2.5%) and local/locoregional recurrence (4.2%) were low, comparing favorably with previously published results for patients with CGEJ undergoing TTE with curative intent [8, 9, 16, 30–32].

We need to qualify our very favorable results by noting that they not only be attributed to the surgical technique but also to the neoadjuvant therapy administered to all patients with T3–T4 tumors, where tumor reduction likely limited the incomplete local resection risks [30].

Nevertheless, the main controversy remains the optimal extent of lymphadenectomy. A median of 17.5 lymph nodes was examined from each specimen, including mediastinal lymph nodes, exceeding the results of published series of THE [8, 17, 33] and achieving comparable lymphadenectomy than TTE [11, 14, 16, 24]. The only randomized study conducted failed to demonstrate a significant association between the higher lymph node yield in the TTE group and an increased 5-year overall survival rate [9]. Furthermore, in a post hoc analysis of the randomized CROSS-II trial, the total number of resected nodes was only correlated with improved OS after surgery alone, whereas the number of invaded lymph nodes was correlated with survival in both groups [16]. Currently, there is increased consensus that this criterion is a prognostic factor after the resection of adenocarcinoma of the esophagus or esophagogastric junction, as is the case with the lymph node ratio [10, 13, 32, 34]. In our study, just over 50% of patients had evidence of nodal metastases after neoadjuvant therapy, which is significantly less than the clinical N-stage (80%). This finding suggests that tumoral regression occurred at the nodal level after neoadjuvant therapy. Indeed, a study demonstrated that after neoadjuvant therapy, not only did the frequency of lymph node metastases decrease but also there was a change in their distribution [35].

Based on a multivariate analysis of the entire population, the only predictive factor of survival in our study was the tumor stage. The 5-year OS rate of nearly 65% observed in this study is in stark contrast to that previously reported [8, 14, 24–26, 32, 33]. Recent studies assessing survival after neoadjuvant treatment and resection for adenocarcinoma of the esophagus reported 3-year OS rates of 53.9–57.4% [30] and 5-year rates of 33–44.3% [31, 36] regardless the surgical technique. The only study focusing on locally advanced tumors included patients without neoadjuvant treatment and epidermoid tumors. The 5-year survival rates were 35% after TTE and 19% after THE [12].

This highlights the difficulty of comparing our results with those of previous studies due to the heterogeneity in disease stage and preoperative treatment.

While the surgical technique used was standardized and applied uniformly, different neoadjuvant regimens were used during the study period. This problem is inevitable when assessing the long-term survival for this type of pathology [30, 31, 36], as practices have evolved considerably following the publication of multicenter randomized trials [4, 19, 20].

Our study is limited by its monocentric non-comparative design. However, the prospective evaluation, large sample of patients with homogeneous tumor characteristics, and lack of exclusion criteria allow us to propose some relevant points that may add to the debate regarding the optimal therapy for CGEJ.

## Conclusions

THE meets the oncological surgical quality criteria both for the extent of resection and quality of lymphadenectomy in locally advanced CGEJ tumors after neoadjuvant treatment. Furthermore, it shows excellent oncological outcomes in terms of survival. This technique can be recommended for all patients with CGEJ, regardless of the tumor stage and patients’ comorbidities.

In the era of neoadjuvant therapy and minimally invasive surgery, oncologic safety after THE remains to be proven in large randomized controlled studies.

## Data Availability

The data that support the findings of this study are available from the corresponding author, [HM], upon reasonable request.
