# Correction to: Transhiatal esophagectomy as a treatment for locally advanced adenocarcinoma of the gastroesophageal junction: postoperative and oncologic results of a single-center cohort: THE for locally advanced GEJC

**DOI:** 10.1186/s12957-022-02577-3

**Published:** 2022-04-01

**Authors:** Hélène Meillat, Vincent Niziers, Christophe Zemmour, Jacques Ewald, Jean-Philippe Ratone, Slimane Dermeche, Jérôme Guiramand

**Affiliations:** 1 Department of Digestive Surgical Oncology, Institut Paoli Calmettes, 232 Boulevard de Sainte Marguerite, 13009 Marseille, France; 2 Department of Clinical Research & Investigation, Biostatistics & Methodology Unit, Paoli Calmettes Institute, Aix Marseille University, INSERM, IRD, SESSTIM, Marseille, France; 3 Digestive Endoscopy Unit, Paoli Calmettes Institute, Marseille, France; 4 Department of Medical Oncology, Institut Paoli Calmettes, Marseille, France

**World J Surg Oncol 20, 70 (2022)**

**https://doi.org/10.1186/s12957-022-02537-x**

Following the publication of the original article [1], the author reported that in the authors name, the given name should be the surname and vice versa. Moreover, in the article title, “THE for locally advanced GEJC” should be a subtitle.

The original article has been updated.
